# The antimicrobial effects of the alginate oligomer OligoG CF-5/20 are independent of direct bacterial cell membrane disruption

**DOI:** 10.1038/srep44731

**Published:** 2017-03-31

**Authors:** Manon F. Pritchard, Lydia C. Powell, Saira Khan, Peter C. Griffiths, Omar T. Mansour, Ralf Schweins, Konrad Beck, Niklaas J. Buurma, Christopher E. Dempsey, Chris J. Wright, Philip D. Rye, Katja E. Hill, David W. Thomas, Elaine L. Ferguson

**Affiliations:** 1 Advanced Therapies Group, Oral and Biomedical Sciences, School of Dentistry, College of Biomedical and Life Sciences, Cardiff University, Heath Park, Cardiff, UK; 2 Department of Pharmaceutical, Chemical and Environmental Sciences, Faculty of Engineering and Science, University of Greenwich, Medway Campus, Central Avenue, Chatham Maritime, UK; 3 Institut Laue-Langevin, DS/LSS group, 6 rue Jules Horowitz, 38042 Grenoble Cedex 9, France; 4 Physical Organic Chemistry Centre, School of Chemistry, Cardiff University, Cardiff, UK; 5 School of Biochemistry, Biomedical Sciences Building, University Walk, Clifton, BS8 1TD, UK; 6 Centre for NanoHealth, Systems and Process Engineering Centre, College of Engineering, Swansea University, Swansea, UK; 7 AlgiPharma AS, Sandvika, Norway

## Abstract

Concerns about acquisition of antibiotic resistance have led to increasing demand for new antimicrobial therapies. OligoG CF-5/20 is an alginate oligosaccharide previously shown to have antimicrobial and antibiotic potentiating activity. We investigated the structural modification of the bacterial cell wall by OligoG CF-5/20 and its effect on membrane permeability. Binding of OligoG CF-5/20 to the bacterial cell surface was demonstrated in Gram-negative bacteria. Permeability assays revealed that OligoG CF-5/20 had virtually no membrane-perturbing effects. Lipopolysaccharide (LPS) surface charge and aggregation were unaltered in the presence of OligoG CF-5/20. Small angle neutron scattering and circular dichroism spectroscopy showed no substantial change to the structure of LPS in the presence of OligoG CF-5/20, however, isothermal titration calorimetry demonstrated a weak calcium-mediated interaction. Metabolomic analysis confirmed no change in cellular metabolic response to a range of osmolytes when treated with OligoG CF-5/20. This data shows that, although weak interactions occur between LPS and OligoG CF-5/20 in the presence of calcium, the antimicrobial effects of OligoG CF-5/20 are not related to the induction of structural alterations in the LPS or cell permeability. These results suggest a novel mechanism of action that may avoid the common route in acquisition of resistance via LPS structural modification.

Multi-drug resistant (MDR) bacteria represent a major global health challenge with soaring morbidity and mortality^1^. Furthermore, as the acquisition of resistance now supersedes the rate of development of new antibiotics, the need for novel antimicrobial therapies is urgent^2^. OligoG CF-5/20 is a low molecular weight (Mn 3,200 g/mol) alginate derived from the stem of the seaweed *Laminaria hyperborea*^3^. OligoG CF-5/20 potentiates the effect of conventional antimicrobials against a range of bacteria^3^ and fungi^4^, and disrupts biofilm formation of MDR pathogens both *in vitro* and *in vivo*^5^. However, the mechanism by which OligoG CF-5/20 exerts its antimicrobial effects is still unclear. OligoG CF-5/20 has been proven safe for human clinical use and has completed Phase 2b clinical studies (NCT02157922; NCT02453789) as an inhalation therapy for cystic fibrosis (CF) patients.

CF is an autosomal recessive disorder causing an imbalance in ion exchange across the respiratory airway^6^, leading to thick mucus stasis, which is ultimately chronically colonised by opportunistic pathogens, principally *Pseudomonas aeruginosa*^7^. CF patients are especially at risk of harbouring MDR bacteria due to the presence of chronic lung infection, compounded by intense and frequent use of multiple antibiotics from childhood. Having previously demonstrated that OligoG CF-5/20 treatment was effectively associated with disruption of planktonic and biofilm growth of *P. aeruginosa*^3^,^8^,^9^, and that this effect was independent of an interaction with the *P. aeruginosa mexAB*-*oprM* efflux pump system^3^, this study sought to investigate whether OligoG CF-5/20 exerts its antibiotic potentiation effects (up to 512-fold) via direct interaction with the bacterial cell.

Whilst many antimicrobials act on the biosynthetic pathways of growing cells, the bacterial membrane represents an important target in the treatment of quiescent non-replicating bacteria in recalcitrant infection such as in the CF lung^10^. A number of agents have been developed that modulate changes in the bacterial membrane directly, via alterations in NADH_2_ and ATP synthase, and indirectly, via generation of lethal reactive oxygen species and nitric oxide in the bacterial membrane. Membrane-active antibiotics, such as the polymyxins, including colistin (polymyxin E) and polymyxin B, and amphipathic antimicrobial peptides, such as RTA3^11^, act synergistically with other drugs to enhance their internalisation and access to intracellular targets^12^.

OligoG CF-5/20 modifies the surface charge of *P. aeruginosa*, inducing cellular aggregation and a reduction in bacterial motility^8^; changes which are associated with decreased mechanical strength of the biofilm structure^9^. A combination effect of OligoG CF-5/20 and the antimicrobial triclosan against the oral pathogens *Streptococcus mutans* (Gram-positive) and *Porphyromonas gingivalis* (Gram-negative) led to a decrease in attachment to surfaces such as titanium^13^. Following the reported interaction of OligoG CF-5/20 with both these Gram-negative and Gram-positive pathogens, a greater understanding of the interaction of the oligosaccharide with the cell wall was sought. Gram-positive bacteria have a single lipid membrane surrounded by a 30–100 nm thick peptidoglycan/lipoteichoic acid cell wall^14^, which is tightly cross-linked by inter-peptide bridges and has a phosphoryl group located in the substituent teichoic and teichuronic acid residues, and un-substituted carboxylate groups (Fig. 1a). In comparison, Gram-negative bacteria have a very thin, loosely cross-linked peptidoglycan, which is sequestered within the periplasmic space, between the inner and outer lipid membranes. Phosphoryl and 2-keto-3-deoxyoctonate carboxylated groups of lipopolysaccharide (LPS) are found in the outer leaflet of the outer membrane (Fig. 1b)^15^. Cell-surface oligosaccharides such as the hydrophilic *O*-antigen component of LPS in Gram-negative bacteria^16^ also play a role in facilitating biofilm attachment. The highly polyanionic nature of LPS maintains the integrity of the outer membrane which is linked electrostatically by divalent cations such as Ca^2+^ 17. The outer membrane of Gram-negative bacteria is selectively resistant to noxious agents due to its effective permeability barrier function (enabling hydrophobic drugs to diffuse across the lipid bilayer, whilst small hydrophilic drugs use the porins to gain access to the cell). Both Gram-positive and Gram-negative bacteria have an overall negative electrostatic surface charge.

Here we present a range of nanoscale techniques to analyse the interaction of OligoG CF-5/20 with components of the bacterial cell wall and membrane permeability, in particular to *P. aeruginosa*. Detailed nanoscale analysis of the interaction of drugs with the bacterial cell can be used to enhance our understanding of the mechanism of action involved in antimicrobial therapy^18^. Atomic force microscopy (AFM) is fast becoming a common tool for analysing nanostructures^19^ and has been used to study the effect of antimicrobial agents on planktonic cells^8^,^20^,^21^ and bacterial biofilms^22^,^23^ as well as a range of MDR Gram-negative organisms^24^,^25^. Cellular surface charge can be analysed using electrophoretic light scattering (ELS), now a standard method for measuring the zeta potential^26^. ELS is often used to explore mechanisms of bacterial adhesion and aggregation to biophysical host tissues and biomaterial substrates^27^,^28^,^29^. Small-angle neutron scattering (SANS) has previously been used to characterise the shape and interaction of bio-macromolecules such as antibiotics and polymers with key bacterial cell wall components, such as LPS^30^. Circular dichroism (CD) spectroscopy has been extensively used to characterise antimicrobial peptides^31^ and analyse their interaction with the bacterial cell wall^32^,^33^. Here CD was used to monitor whether LPS interacts with OligoG CF-5/20 via its carboxyl groups that show intense Cotton effects near 200 and 215 nm^34^. Isothermal titration calorimetry has also previously been employed to elucidate the mechanisms by which novel antimicrobials interact with the cell surface target, LPS^35^,^36^.

## Results

### Comparison of bacterial cell wall and the effect of OligoG CF-5/20

**Figure 1:**
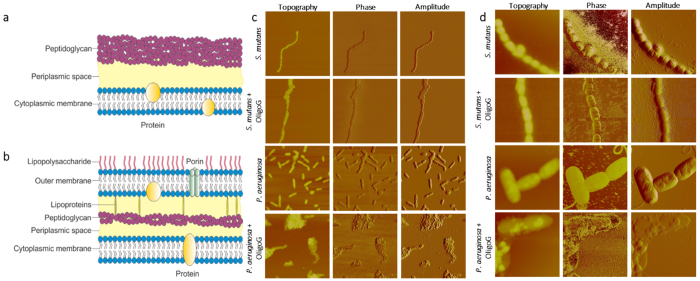
Comparison of the effect of OligoG CF-5/20 on Gram-positive and Gram-negative bacteria. Diagram representing the cell wall of (**a**) Gram-positive and (**b**) Gram-negative bacteria. Atomic force microscopy (AFM) images of *S. mutans* ± 7 mg/ml OligoG CF-5/20 and *P. aeruginosa* ± 5 mg/ml OligoG CF-5/20 at (**c**) 20 μm^2^ and (**d**) 5 μm^2^ (*S. mutans*) and 4 μm^2^ (*P. aeruginosa*).

AFM images of Gram-positive *S. mutans* and Gram-negative *P. aeruginosa* treated with OligoG CF-5/20 (7 and 5 mg/ml respectively), showed cellular aggregation, which was not evident in the untreated bacteria (Fig. 1c). OligoG CF-5/20 appeared to surround the cell walls of *P. aeruginosa* following a centrifugation step, prior to imaging. However, while Gram-positive *S. mutans* demonstrated cellular clumping, OligoG CF-5/20 was not visible around the cell surface at the nanoscale level upon exposure to centrifugation, when compared to *P. aeruginosa* (Fig. 1d).

### Effect of OligoG CF-5/20 on cell permeability

**Figure 2:**
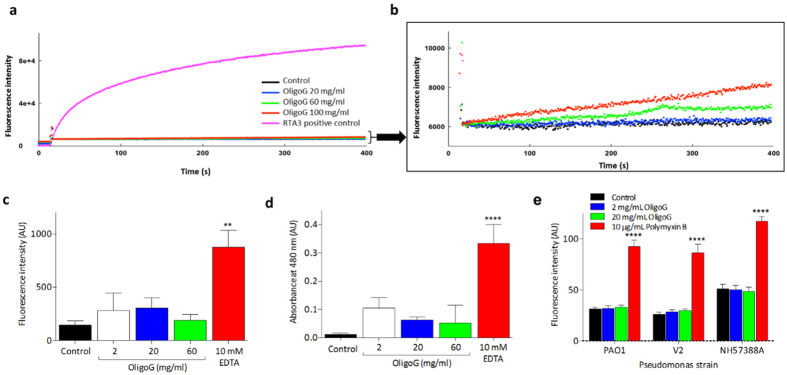
The effect of OligoG CF-5/20 on bacterial cell membrane permeabilisation. Cell permeability assay showing (**a**) release of carboxyfluorescein (Cbfl) from single lamellar liposomes composed of egg PC:PG (80:20) in the presence of RTA3 (0.5 μM; positive control) or OligoG CF-5/20 at 20–100 mg/ml (**b**) zoomed-in graph of OligoG CF-5/20 only data. Internalisation of (**c**) propidium iodide (PI) and (**d**) nitrocefin (NFN) by PAO1 compared to EDTA (positive) control (1 mM-20 mM). (**e**) Internalisation of 1-N-phenylnaphthylamine (NPN) dye by *P. aeruginosa* strains; PAO1, V2 and NH57388A ± 2–20 mg/ml OligoG CF-5/20 and polymyxin B (positive control). (AU = arbitrary units). (Data represents mean ± SD; **p < 0.01 and ****p < 0.0001 compared to control; n = 3).

Having demonstrated that OligoG CF-5/20 causes cellular aggregation in Gram-negative bacteria, with OligoG CF-5/20 surrounding the cell walls, the ability of the alginate to permeabilise both simulated (liposomes) and real cell membranes, with propidium iodide (PI), nitrocefin (NFN) and 1-N-phenylnaphthylamine (NPN), was studied using conventional permeability assays. Initial studies using carboxyfluorescein-loaded unilamellar liposomes showed that, unlike RTA3 under these conditions, an amphipathic antimicrobial peptide, OligoG CF-5/20 had virtually no membrane perturbing effects (Fig. 2a), although it did produce a slight dose-dependent increase in release of trapped dye (Fig. 2b). Similar results were obtained in vesicles composed of PC:PG at a ratio of 50:50 (data not shown).

Correspondingly, in an *in vitro* model of membrane permeabilisation in *P. aeruginosa* PAO1, neither PI (Fig. 2c) nor NFN (Fig. 2d) were able to enter the cytoplasm and periplasmic space, respectively, in the presence of OligoG CF-5/20. As OligoG CF-5/20 is able to bind Ca^2+^ we also compared its effect to the chelating agent, ethylendiaminetetraacetic acid (EDTA), a chelator which effectively permeabilises the bacterial outer membrane of PAO1, allowing internalisation of both the dyes. In a final evaluation of the ability of OligoG CF-5/20 to enhance cell permeability, internalisation of NPN dye by three *P. aeruginosa* strains was assessed. As seen in the other assays, OligoG CF-5/20 (up to 20 mg/ml) did not promote partitioning of NPN into bacterial cell membranes, which was clearly evident in the presence of the positive control, polymyxin B (Fig. 2e).

### Effect of OligoG CF-5/20 under various osmolyte conditions

**Figure 3:**
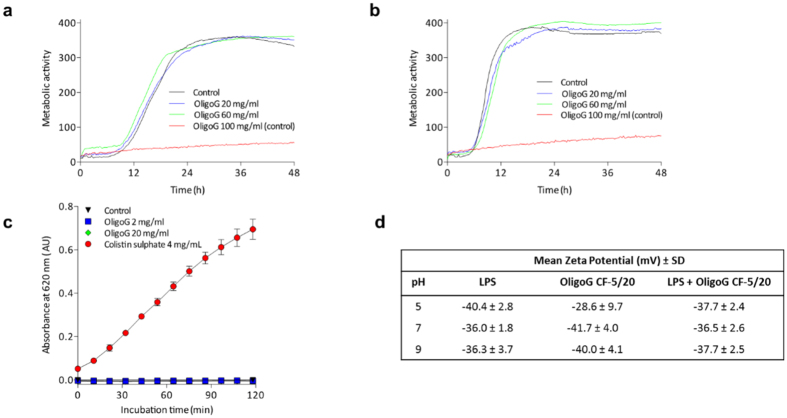
Effect of OligoG CF-5/20 on *Pseudomonas aeruginosa* PAO1 under various osmolyte conditions and on lipopolysaccharide (LPS from *P. aeruginosa*). Biolog metabolomic osmolyte assay (PM9) representing PAO1 ± 20–100 mg/ml OligoG CF-5/20 (**a**) 4% urea (**b**) 20 mM sodium benzoate pH 5.2 (48 h). (**c**) Precipitation of LPS from *P. aeruginosa* (5 mg/ml) ± 2–20 mg/ml OligoG CF-5/20 or 4 mg/ml colistin sulphate (positive control). (Data represents mean ± SD, n = 3). (**d**) Mean zeta potential measurements of 2 mg/ml OligoG CF-5/20 and 10 mg/ml LPS in 0.01 M NaCl buffer at pH 5, 7 and 9.

PAO1 (48 h) showed no changes in growth in response to ionic/osmotic stress in the presence of OligoG CF-5/20 (20–60 mg/ml) under all conditions tested, including 4% (w/v) urea (Fig. 3a) and 20 mM sodium benzoate pH 5.2 (Fig. 3b).

### Surface charge and aggregation of LPS in the presence of OligoG CF-5/20

Having eliminated the possibility of cell permeabilising effects, the direct interaction of OligoG CF-5/20 with LPS was studied. First, LPS aggregate formation with OligoG CF-5/20 (or colistin sulphate as a positive control) was studied by measuring change in turbidity over time. Turbidity remained unaltered in the presence of OligoG CF-5/20 (up to 20 mg/ml), although significant differences in turbidity were observed with the positive control, colistin sulphate, which rapidly formed aggregates with LPS (Fig. 3c). Surface charge (zeta potential) of pseudomonal LPS alone became slightly less negative as the pH increased from pH5 to pH7 and pH9 (−40.4 mV, −36.0 mV and −36.3 mV, respectively; Fig. 3d) with only a small overall change in charge over this pH range (4.1 mV). In contrast, the zeta potential of OligoG CF-5/20 alone showed a greater change in charge (11.4 mV) over the pH range tested, being significantly less negative at pH 5 compared to pH 7 and 9 (−28.6 mV, −41.7 mV and −40.0 mV, respectively; p < 0.05). However, when OligoG CF-5/20 and LPS were combined, there was no pH-dependent change in surface charge interaction when compared to LPS alone.

### Structural interactions of OligoG CF-5/20 with LPS

**Figure 4:**
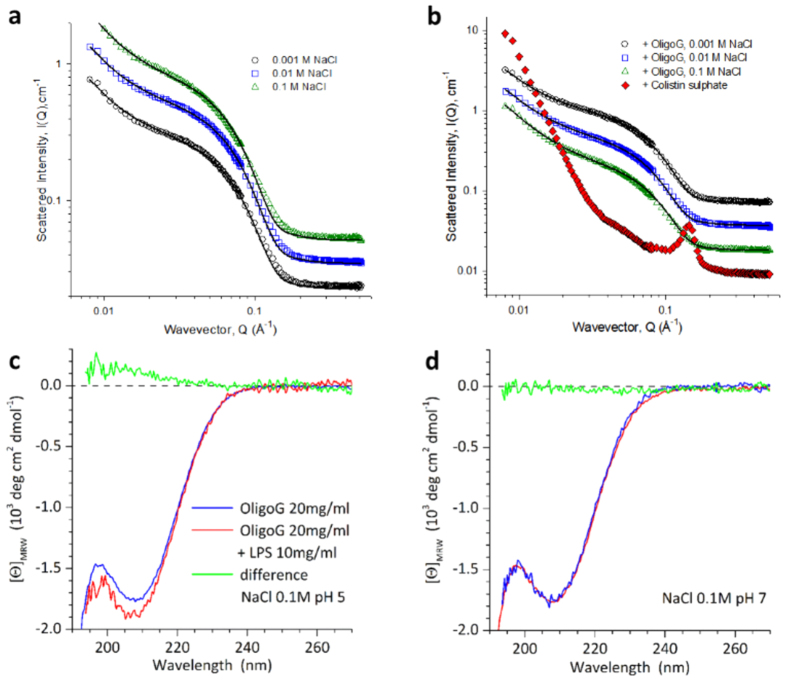
Structural analysis of LPS ± OligoG CF-5/20. Small-angle neutron scattering from LPS (10 mg/ml) in D_2_O containing 0.001 to 0.1 M NaCl at pH 7 (**a**) alone and (**b**) following incubation (3 h at 37 °C) with OligoG CF-5/20 (20 mg/ml). Circular dichroism spectra of LPS (10 mg/ml) and OligoG CF-5/20 (20 mg/ml) in 0.1 M NaCl at (**c**) pH 5 and (**d**) pH 7 were recorded at 37 °C. OligoG CF-5/20 spectra in the absence and presence of 10 mg/ml LPS are shown in blue and red, respectively; the difference of the spectra is shown in green.

SANS experiments of LPS showed significant scattering intensity I(Q) as a function of the wave-vector, Q, which varied subtly at low Q as a function of ionic strength (Fig. 4a) but was largely unaltered by pH (see Supplementary Fig. S1). Pre-incubation of LPS with OligoG CF-5/20 had no effect on scattering intensity. However, when LPS was pre-incubated with a positive control, colistin sulphate, a pronounced increase in scattering intensity at low Q was apparent, indicating larger structures. Additionally, two peaks appeared at Q = 0.06 and 0.12 Å^−1^, demonstrating a regular structure of stacked interfaces (Fig. 4b). The most striking observation from the SANS experiment, is that the scattering did not change with ionic strength or the addition of OligoG CF-5/20. Indeed, when analysing the data in terms of a mixture of vesicles and micelles, not surprisingly, the parameters required to fit the data were also largely constant. The balance of the vesicular to micellar components was also invariant with both variables. Noteworthy is the comparison of the radius in the micellar term (22 Å), presumably corresponding to the extended length of the LPS molecule, *versus* the thickness of the vesicular lamellae, 46 Å, which one would expect to be double the extended length.

CD spectra recorded under similar conditions showed no apparent conformational changes that could indicate a specific LPS:OligoG CF-5/20 interaction. The spectra resulting after mixing simply corresponded to the addition of the individual signals (Fig. 4c,d). This was also found when varying the OligoG CF-5/20 concentration between 2 to 20 mg/ml (data not shown). An ionic interaction of LPS with the carboxylates of OligoG CF-5/20 would be expected to reduce the CD amplitude at ~215 nm and could induce a red-shift of the signal, as observed for the interaction with calcium ions^37^.

**Figure 5:**
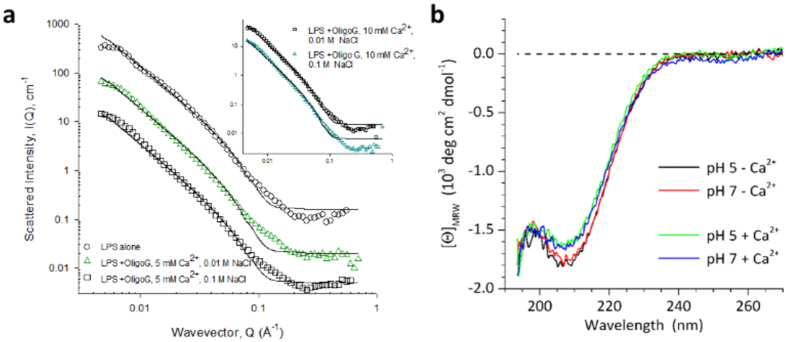
LPS analysis in the presence of divalent cations. (**a**) Small-angle neutron scattering (SANS) analysis of LPS (10 mg/ml) and OligoG CF-5/20 (20 mg/ml) in the presence of 5 or 10 mM Ca^2+^ in 0.01 or 0.10 M NaCl at pH 7. (**b**) Circular dichroism spectra of 20 mg/ml OligoG CF-5/20 in the presence of 10 mg/ml LPS in 0.10 M NaCl were recorded in the presence and absence of 5 mM Ca^2+^ at pH 5 and pH 7 at 37 °C; buffer spectra including LPS were subtracted.

The SANS data also showed that Ca^2+^ at 5 and 10 mM had no effect on the LPS structure over the length scale probed (Fig. 5a). The raw data follows a rather less curved form, with just a very weak oscillation. The best fit here was found to be a given by the simple unilamellar vesicle with a radius slightly larger than the previous case, but with a similar thickness, at least within experimental error, *i.e*. there was no clear evidence of co-existing smaller micelles. The key parameters for all SANS experiments are presented in Table 1. Also, CD spectra for LPS/OligoG CF-5/20 interactions measured with and without 5 mM Ca^2+^ at pH 5 and pH 7 showed no significant differences (Fig. 5b).

### Biomolecular interactions of OligoG CF-5/20 and LPS

Initially, ITC was employed to record the heat effects of OligoG CF-5/20 (20 mg/ml, ~6.25 mM) dilutions. The dilution heat effects of OligoG CF-5/20 in the presence of 1 mM EDTA showed only a limited decrease, suggesting that the aggregation state of OligoG CF-5/20 did not change with increasing concentration. In the presence of 1 mM Ca^2+^, however, the dilution heat effects were not constant and followed the typical pattern for self-aggregating compounds^38^, which was strongly suggestive of OligoG CF-5/20 aggregation in the presence of added Ca^2+^. (Data obtained in the presence of 1 mM CaCl_2_ was comparable to titrations in the presence of 1 mM EDTA and 2 mM CaCl_2_, see Supplementary Fig. S2).

**Figure 6:**
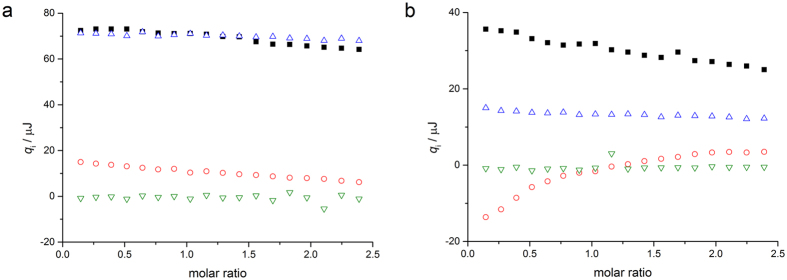
Interaction between OligoG CF-5/20 and LPS. (**a**) Heat effects per injection (*q*_i_) for the titration of 20 mg/ml OligoG CF-5/20 into 10 mg/ml LPS (▪), 20 mg/ml OligoG CF-5/20 into buffer (o), buffer into 10 mg/ml LPS (Δ), and buffer into buffer (∇) at 37 °C (buffer is 20 mM phosphate pH 7, 100 mM NaCl, 1 mM EDTA). (**b**) Heat effects per injection (*q*_i_) for the titration of 20 mg/ml OligoG CF-5/20 into 10 mg/ml LPS (▪), 20 mg/ml OligoG CF-5/20 into buffer (o), buffer into 10 mg/ml LPS (Δ), and buffer into buffer (∇) at 37 °C (buffer is 20 mM phosphate pH 7, 100 mM NaCl, 1 mM EDTA).

Further studies were conducted to determine the interaction between OligoG CF-5/20 and LPS, both in the presence of 1 mM EDTA or CaCl_2_ and when combining 1 mM EDTA and 2 mM CaCl_2_. In the presence of EDTA alone, (i.e. in the absence of free Ca^2+^) the heat effects for injection of OligoG CF-5/20 into LPS did not deviate significantly from the combined heat effects for the reference dilution experiments (Fig. 6a). This observation suggested that in the absence of free Ca^2+^, OligoG CF-5/20 and LPS do not interact at the concentrations used in these experiments. Contrastingly, in the presence of 1 mM added CaCl_2_, (or 1 mM EDTA and 2 mM CaCl_2_), the heat effects observed for injection of OligoG CF-5/20 into LPS were markedly different from the combined heat effects for the reference dilution experiments. In particular, whilst de-aggregation of OligoG CF-5/20 upon dilution was exothermic (*vide supra*), interaction of OligoG CF-5/20 with LPS was endothermic, strongly suggesting that OligoG CF-5/20 and LPS interact in the presence of calcium (Fig. 6b). The lack of a sigmoidal shape to the enthalpogram suggested, however, that the interaction was weak^39^.

## Discussion

OligoG CF-5/20 is a new antimicrobial therapy, demonstrating promising results across the microbial kingdom in both eubacteria and yeasts. Effective synergistic enhancement of current antimicrobials has previously been demonstrated in both Gram-positive^13^ and Gram-negative bacteria^3^. This study focused on the nanoscale interaction of OligoG CF-5/20 with the Gram-negative cell surface, following strong, irreversible binding to the cell wall after centrifugation. OligoG CF-5/20 has previously been shown to remain bound to the pseudomonal cell surface, leading to cellular aggregation, even following exposure to hydrodynamic shear^8^.

Structural analysis of pseudomonal biofilms has previously indicated that OligoG CF-5/20 treatment was associated with increased water channels as demonstrated by scanning electron and confocal laser scanning microscopy studies^3^ and a decrease in biofilm mechanical strength as shown by rheological analysis and AFM force measurements^9^. Clear differences were seen at the nanoscale level, showing significantly greater surface interaction of OligoG CF-5/20 with the Gram-negative cell wall of *P. aeruginosa*, which remained attached to the *P. aeruginosa* cell wall, and resisted hydrodynamic shear^8^. A previous study quantified the alteration in PAO1 surface charge and aggregation using electrophoretic and dynamic light scattering, and confirmed the irreversible binding between OligoG CF-5/20 and the cell surface^8^. No change in surface charge was seen with the Gram-positive *S. mutans* when treated with OligoG CF-5/20 following hydrodynamic shear (Supplementary Fig. S3). The presence of a dense layer of LPS is unique to Gram-negative bacteria and provides an effective (although selective) permeability barrier^40^. We hypothesised that OligoG CF-5/20 may directly interact with LPS to reduce biofilm formation and further experiments were conducted solely in Gram-negative *P. aeruginosa* strains.

Permeabilisation studies in *P. aeruginosa* demonstrated that the cellular membrane changes induced by membrane-active agents, EDTA, and polymyxin B and the synthetic peptide RTA3, were virtually absent in the presence of OligoG CF-5/20 (Fig. 2). Similarly, metabolomic-profiling studies demonstrated that bacterial growth with OligoG CF-5/20 was unaffected by changes in osmotic/ionic conditions (Fig. 3a,b). The lack of permeabilisation was supported by growth assays that showed only bacteriostatic activity with OligoG CF-5/20^3^. Nevertheless, this could be advantageous as the development of many membrane-active antimicrobial agents has been hampered by formulation difficulties and non-specific permeabilisation/toxicity concerns^41^. The putative membrane effect with OligoG CF-5/20 is supported by the absence of resistance to the drug during prolonged serial passage^3^.

Previous force-curve measurements on *P. aeruginosa* PAO1 biofilms showed a decrease in Young’s modulus when treated with OligoG CF-5/20 (20–100 mg/ml)^9^, which correlated with an alteration (3.1–6.0 mV decrease) in surface charge^8^. However, these results were not reflected in the LPS ELS analysis in this study (Fig. 3d). Conversely, previous studies have noted that ELS and AFM analysis for LPS may not always correlate, as bacterial adhesion can vary depending on LPS chain length^42^.

SANS and CD experiments were employed to gain a greater understanding of the interaction of OligoG CF-5/20 with Gram-negative cell wall components at the nanoscale. SANS has previously been used to analyse the structure of LPS and its derivatives, highlighting the different chemotypes of LPS (rough and smooth) and the importance of temperature control^43^,^44^,^45^. In these studies, colistin was used as a positive control due to its known ability to bind and neutralise bacterial LPS^46^ displacing cell wall-stabilising divalent cations in the outer membrane^47^. Previous studies have investigated the direct interaction of colistin with LPS from *Escherichia coli* using turbidity, CD and SANS experiments^30^. Pseudomonal LPS aggregates demonstrated a high scattering intensity, *I(Q*) as a function of the wave-vector, *Q*, which was in line with the size, shape and distribution of *E. coli* LPS observed previously^30^. These studies, along with others^48^, have demonstrated that at neutral pH, LPS forms structures that are hundreds of nm in size but with a lamellar organization and bilayer thickness of ~5 nm. Similarly, the emergence of two peaks when LPS was treated with colistin, was accompanied by a pronounced increase in scattering intensity at low *Q*, as seen with *E. coli* LPS and colistin^30^. The conformation of the LPS was unaltered by OligoG CF-5/20 at all pHs and salt concentrations tested (Fig. 4a,b), which are comparable to those previously reported in PAO1 zeta potential analysis^8^. Similar to the current study, CD spectra of LPS-colistin mixtures showed no indication of conformational changes, but only changes that could be interpreted as simple additive effects (Fig. 4c,d).

Salt concentration and pH have a fundamental effect on bacterial surface charge, and these parameters are altered in CF patients during an exacerbation and when in remission. Several studies have shown a broad variation of acidity in the lung environment of a CF patient (pH 5–6) which is lowered during an exacerbation, while normal lung fluid pH is ~7^49^,^50^. Similarly, the chloride concentration of CF lung fluid is abnormally high, due to defective chloride channels and/or chronic lung infection and inflammation with the chloride concentration of tracheal and bronchial airway surface fluid increasing from 85 ± 54 mM in healthy individuals to 129 ± 79 mM in CF patients^51^. Encouragingly, these studies demonstrate that OligoG CF-5/20 did not cause any conformational changes in LPS at physiological pH and salt concentrations.

The discovery that the antimicrobial activity of OligoG CF-5/20 does not depend on cell permeabilisation is promising, since this is the type of mechanism of action that is commonly found to be the cause of antibiotic resistance^52^. Colistin is increasingly being used for the treatment of MDR Gram-negative bacterial infections^53^ and is the most commonly inhaled antibiotic treatment in CF^54^. The recent emergence of colistin resistance is of major concern^55^ and is linked to LPS modification^56^ possibly as a result of complete loss of LPS in strains such as *Acinetobacter baumannii*^57^. LPS modification mediated by the *pmr* operon is also known to enhance colistin resistance^58^. Recently, OligoG CF-5/20 has been shown to potentiate the effect of colistin against MDR pseudomonal pathogens, leading to a 128-fold reduction in the Minimum Biofilm Eradication Concentration value^5^.

A significant alteration in divalent cation levels has been reported in the CF lung (102 mg/l Ca^2+^) compared to 45 mg/l in a healthy control^59^. The effect of divalent metal ions in maintaining LPS structure has been well documented in the literature, with their depletion found to lead to a distinct outer membrane structure with an exposed peptidoglycan surface layer, and increased cell permeability^40^. Colistin is believed to electrostatically bind to the anionic phosphate groups on the LPS lipid A core, leading to displacement of the divalent cations which bridge the lipid A molecules and maintain cell wall integrity^47^. Previous *in vivo* MDR bacterial biofilm models of infection have demonstrated a dramatic reduction in biofilm growth (2.5-log fold) when OligoG CF-5/20 was administered intra-tracheally^5^.

Gram-negative bacteria do not exhibit membrane perturbation following binding. Instead we hypothesise that the anti-biofilm effects of OligoG CF-5/20 are mediated via interaction with the bacterial matrix of extracellular polysaccharide substance (EPS), removing this barrier and allowing more effective interaction of colistin with the bacterial cell wall. This mechanism of action, by EPS disruption, may be promising in the treatment of MDR biofilm infections^60^.

Due to its overall negative charge, OligoG CF-5/20 did not self-aggregate in the absence of free Ca^2+^. However, Ca^2+^-induced self-aggregation can overcome the electrostatic repulsion between individual molecules, via the formation of salt bridges. In fact, previous calorimetric studies have shown similar Ca^2+^-mediated aggregation of alginates via so-called ‘egg-box’ dimers^61^. Unsurprisingly, the interaction between OligoG CF-5/20 and LPS was also found to be mediated by Ca^2+^, but the interaction was not sufficiently strong enough to allow analysis in terms of a simple binding model (stoichiometry and affinity). The ITC studies, concluding that only a weak (Ca^2+^-dependent) interaction between OligoG CF-5/20 and LPS occurred, alongside the physical analysis (ITC/SANS/CD spectroscopy), all suggested that OligoG CF-5/20 did not significantly alter the structure of LPS. These interactions may, in part, be reflected in the bacterial aggregation observed in AFM.

In summary, OligoG CF-5/20 induced cellular aggregation of both *S. mutans* and *P. aeruginosa*, however, irreversible surface interaction of OligoG CF-5/20 was demonstrated with the Gram-negative cell. No increase in permeability of the membrane was detected when treated with OligoG CF-5/20. OligoG CF-5/20 also did not induce surface charge alterations of the LPS component of the outer membrane, nor did it neutralise or cause aggregation of LPS itself. Subtle changes in LPS conformation were recorded in solution following an increase in salt concentration, however pH had no apparent effect. CD spectra for LPS remained unaltered by OligoG CF-5/20, as did the presence of Ca^2+^. ITC, however, showed a weak Ca^2+^ mediated interaction between OligoG CF-5/20 and LPS. Studies are ongoing to determine the molecular mechanism of action of the antimicrobial properties of OligoG CF-5/20. It is hoped that defining its mode of action will help with the advancement of future applications for this antimicrobial agent, to address the developmental need for new antimicrobial therapies.

## Materials and Methods

### Materials

OligoG CF-5/20 was synthesised as previously described^3^. Materials were obtained from the following companies: deuterium oxide (D_2_O; with 99.9% isotopic purity), LPS (from *Pseudomonas aeruginosa* 10), Triton X-100, carboxyfluorescein (Cbfl), colistin sulphate, polymyxin B, Tris HCl, propidium iodide (PI), 1-*N*-phenylnapthylamine (NPN), ethylenediaminetetraacetic acid (EDTA), sodium fluoride (Sigma-Aldrich, Gillingham, U.K.); sodium chloride (NaCl), calcium chloride (CaCl), hydrochloric acid, sodium hydroxide, acetone (Fisher Scientific, Loughborough, U.K.); phosphate buffered saline (PBS) tablets, tryptic soy broth (TSB), Mueller-Hinton (MH) broth, (Oxoid, Basingstoke, U.K.); nitrocefin, (Calbiochem, Darmstadt, Germany); and egg phosphatidylcholine (PC), phosphatidylglycerol (PG), (Lipid Products, Nutfield, UK).

### Bacteria, Media and Culture Conditions

*Streptococcus mutans* DSM 20523 (ATCC 25175) and *Pseudomonas aeruginosa* strains PAO1, V2 (MDR^3^) and NH57388A (mucoid variant) were grown on blood agar plates or in TSB overnight at 37 °C.

### Atomic Force Microscopy Imaging

Bacterial cultures of *S. mutans* DSM 20523 (72 h) and *P. aeruginosa* (PAO1; 24 h) were grown in TSB at 37 °C. The overnight cultures were washed twice (5,500 *g*, 3 min) in dH_2_O and the bacteria were then incubated in 5–7 mg/ml OligoG CF-5/20 for 20 min. Excess OligoG CF-5/20 was removed (2,500 *g*, 6 min) before resuspending the bacterial cells in dH_2_O and drying on 0.01% poly-L-lysine coated mica plates for imaging. A Dimension 3100 AFM (Bruker) was employed, using tapping mode operation in air (0.8 Hz scan speed).

### Membrane permeability studies

#### Release of carboxyfluorescein dye from a vesicular model of the bacterial membrane

This model, mimicking the Gram-negative bacterial inner membrane, was used to study membrane interactions with OligoG CF-5/20. Small unilamellar liposomes (100 nm) containing carboxyfluorescein (Cbfl; 50 mM) were prepared from egg phospholipids employing the freeze/thaw pressure-extrusion method^11^,^62^ using egg phosphatidylcholine:phosphatidylglycerol at a ratio of 80:20 or 50:50 to mimic the Gram-negative bacterial inner membrane. Cbfl solutions were made by dissolving in 10 mM Tris and adding NaOH to bring the pH to 7.4. Dried phospholipids were hydrated in Cbfl-containing Tris, pH 7.4, freeze-thawing 3 times to support the production of large multilamellar vesicles at a lipid concentration of 10 mg/ml, and then extruding 10 times through two 100 nm pore membranes. The resulting Cbfl-loaded 100 nm small unilamellar vesicles were separated from external Cbfl by passing down a Sephadex G-15 gel filtration column equilibrated with 10 mM Tris, pH 7.4 containing 107 mM NaCl to balance osmotically the internal Cbfl. Release of entrapped Cbfl in the presence of OligoG CF-5/20 (20, 60, 100 mg/ml) was measured in 10 mM Tris-HCl, 107 mM NaCl (pH 7.4) buffer by fluorescence with λ_ex_ = 490 nm and λ_em_ = 520 nm over time (5 min). RTA3^11^ (0.5 μM) and Tris buffer alone were used as positive and negative controls, respectively.

#### Determination of membrane permeabilisation by nitrocefin and propidium iodide uptake of cells

Membrane permeabilisation of PAO1 was quantified by measuring cellular uptake of nitrocefin (a chromogenic β-lactamase substrate)^63^ or propidium iodide (PI)^64^. Cells were grown overnight in TSB, then diluted in PBS (pH 7.4) to an OD_625_ of 0.5. Cells were then washed twice by centrifugation at 3,500 *g* for 10 min at 25 °C to form a pellet, before being resuspended in PBS.

For the nitrocefin assay, OligoG CF-5/20 (0, 20, 60, 100 mg/ml) or EDTA (10 mM) dissolved in PBS (180 μl) were added to the wells of a microtitre plate containing bacterial suspension (10 μl). The plate was sealed with parafilm, incubated at 37 °C for 3 h and centrifuged at 12,000 *g* for 10 min. The supernatant (95 μl) of each well was removed and transferred into the wells of a clean microtitre plate containing nitrocefin (0.5 mg/ml in 5% v/v DMSO, 5 μl). Plates were incubated in the dark at 37 °C for 30 min before measuring the absorbance on a FLUOstar OPTIMA plate reader (BMG LABTEC) at 486 nm (n = 3).

For the PI assay, OligoG CF-5/20 (0, 20, 60, 100 mg/ml) or EDTA (10 mM) dissolved in PBS (140 μl) were added to the wells of a black microtitre plate containing bacterial suspension (10 μl) and PI solution (1.5 mM in PBS, 50 μl). Plates were incubated in the dark at 37 °C for 15 min before measuring fluorescence (λ_ex_ = 480 nm, λ_em_ = 612 nm). Fluorescence intensity was calculated by subtracting the baseline fluorescence of control cells (PBS only) from the total fluorescence of treated cells (n = 3).

#### 1-N-phenylnaphthylamine (NPN) dye assay

Outer membrane permeabilisation of *P. aeruginosa* was quantified by measuring uptake of 1-*N*-phenylnapthylamine (NPN) dye into the bacterial cytoplasmic membrane^65^. Cells were grown overnight in TSB then diluted in PBS, pH 7.4, to an OD_625_ of 0.5. Cells were washed twice by centrifugation at 4,000 rpm for 10 min at 25 °C and resuspended in PBS. Bacterial suspension (100 μl) was added to the wells of a black 96-well plate and mixed with freshly prepared NPN solution (40 μM in 8% v/v acetone; 50 μl) and left to equilibrate at room temperature for 30 min. Solutions (50 μl) of OligoG CF-5/20 (0, 2, 20 mg/ml) or polymyxin B (10 μg/ml) were added to the wells and fluorescence was read immediately using the fluorescent plate reader (λ_ex_ = 350 nm, λ_em_ = 410 nm). Dye uptake was calculated by subtracting the baseline fluorescence of free NPN from the total fluorescence of treated cells (n = 12).

### Effect of OligoG CF-5/20 under various osmolyte conditions

Metabolomic studies were employed to phenotypically screen for the effect of osmolytes on *P. aeruginosa* PAO1 using an osmotic/ionic response assay panel from BIOLOG (Haywood, CA, USA), a 96 well-plate containing different osmolytic conditions^66^. PAO1 was grown overnight on 5% Blood agar at 37 °C. OligoG CF-5/20 (20–60 mg/ml) was dissolved in Innoculating Fluid (IF) 10 (BIOLOG) and incubated at 37 °C for 20–30 min on a roller mixer until dissolved. The remaining ingredients (inoculating fluid base, dye mix, cells and water) were added according to the manufacturers’ instructions prior to loading onto the BIOLOG PM09 plate (100 μl/well). OligoG CF-5/20 and PAO1 only controls were also included. Plates were incubated in an Omnilog incubator at 37 °C for 120 h. As there was no change of activity after this time, results were taken at 48 h. Metabolic activity was analysed colorimetrically by measuring reduction of tetrazolium dye to insoluble formazan (purple colour) by cell respiration^67^ (n = 2). An OligoG CF-5/20 only control (≥60 mg/ml) was included to confirm that no colometric changes in the absence of PAO1 occurred.

### Electrophoretic light scattering (zeta potential) measurements

The zeta potential of pseudomonal LPS (10 mg/ml) in the absence and presence of OligoG CF-5/20 (2 mg/ml) was determined using a Zetasizer Nano ZS (Malvern Instruments) with disposable capillary cells (DTS1061 Malvern Instruments) in 0.01 M NaCl, pH 5, 7 and 9, at 25 °C (n = 10).

### Turbidity assay

A turbidimetric assay was used to measure the binding of OligoG CF-5/20 to LPS resulting from the precipitation of aggregates and increased turbidity. LPS was dissolved in pre-warmed PBS (10 mg/ml, 37 °C, pH 7.4) containing OligoG CF-5/20 (2 and 20 mg/ml) and 200 μl were added to the wells of a 96-well microtitre plate. Control samples contained only LPS dissolved in PBS. Plates were incubated at 37 °C throughout the experiment and absorbance was read at 620 nm at time-points over 2 h. Samples were assayed in triplicate and means calculated.

### Small angle neutron scattering (SANS)

SANS experiments were performed either on the D11 diffractometer at the steady-state reactor source ILL, Grenoble or on SANS2d at the spallation source at ISIS, Oxfordshire. For D11, measurements were performed at a constant neutron wavelength (λ) of 6 Å and sample-detector distances of 1.2 m and 8 m to cover a Q range between 0.008 and 0.5 Å^−1^, whereas for SANS2D, neutron wavelengths spanning 2–14 Å were used to access a Q range 0.02 to 3 Å^−1^. In both cases, the samples were contained in 2 mm path-length, UV-spectrophotometer grade quartz cuvettes (Hellma, U.K.) and mounted in aluminium holders on top of an enclosed, computer-controlled, sample chamber. Sample volumes were around 0.4 cm^3^. All experiments were conducted at 37 °C ± 0.2 °C. Experimental measuring times were approximately 20 min.

To assess the salt- and pH-dependent solution conformation of OligoG CF-5/20, the polymer was dissolved (20 mg/ml) in D_2_O containing 0.001 M, 0.01 M or 0.1 M NaCl at pH 5, 7, or 9. To characterise the OligoG CF-5/20-LPS interaction, LPS (10 mg/ml) was incubated in the absence and presence of OligoG CF-5/20 (2, 20 mg/ml) in D_2_O containing 0.001 M, 0.01 M or 0.1 M NaCl at pH 5, 7, or 9 for 3 h at 37 °C prior to analysis by SANS. Where relevant, calcium chloride was also included to the stated concentration.

All scattering data were (a) normalised for the sample transmission, (b) background corrected using a quartz cell filled with D_2_O, and (c) corrected for the linearity and efficiency of the detector response using the instrument specific software package.

The data without calcium were fitted to a model comprising a coexisting mixture of unilamellar vesicular and spherical micellar structures. However, when combined with calcium, a vesicular structure model, looking at the absolute scattering intensities was utilised^43^.

### Circular dichroism spectroscopy

CD was used to study the interaction of LPS with OligoG CF-5/20. Spectra were recorded on an Aviv 215 spectrophotometer (Aviv Biomedical Inc., Lakewood, NJ) using 0.01cm quartz cuvettes. Samples were prepared by dilution from 25 mg/ml LPS and 50 mg/ml OligoG CF-5/20 stock solutions into the desired buffers and incubated for 3 to 6 h at 37 °C prior to measurements. Spectra were recorded using a 1 nm bandwidth, 0.2 nm intervals with 3 s accumulation time at 37 °C with a dynode voltage less than 500 V. Buffer baselines recorded in the same cell with the same parameters were subtracted. Mean residue weight ellipticities are reported using 194 Da to represent the mass of the carbohydrate units.

### Isothermal Titration Calorimetry (ITC)

Calorimetric titrations were carried out at 37 °C on a MicroCal PEAQ-ITC microcalorimeter (Malvern Instruments Ltd). The instrument was operated applying a reference power of 10 μcal/s, in high feedback mode, stirring the sample cell contents at 750 rpm, with a pre-injection initial delay of 60 s. Freshly prepared solutions of LPS (10 mg/ml, ~0.5 mM) and OligoG CF-5/20 (20 mg/ml, ~6.25 mM) were loaded into the calorimeter sample cell and injection syringe respectively, using the required buffers. Buffers employed in the experiment were 100 mM NaCl, 20 mM NaH_2_PO_4_ (+NaOH adjusted to pH 7) ± the addition of 1 mM EDTA and/or CaCl_2_ (1 mM final free Ca^2+^). All experiments involved an initial injection of 0.4 μL in 0.8 s followed by 18 further injections of 2.0 μL in 4.0 s into the calorimeter sample cell. Injections were spaced by at least 90 s to allow full recovery of the baseline. Raw data was treated using MicroCal PEAQ-ITC Analysis Software (1.0.0.1259) to generate both integrated heat effects per injection (ΔQ) and molar heat effects per injection (ΔH).

### Statistical analysis

The significance of the data was assessed using one-way analysis of variance (ANOVA) followed by Bonferroni’s post hoc test. Statistical significance was set at *p* < 0.05.

## Additional Information

**How to cite this article:** Pritchard, M. F. *et al*. The antimicrobial effects of the alginate oligomer OligoG CF-5/20 are independent of direct bacterial cell membrane disruption. *Sci. Rep.* **7**, 44731; doi: 10.1038/srep44731 (2017).

**Publisher's note:** Springer Nature remains neutral with regard to jurisdictional claims in published maps and institutional affiliations.

## Supplementary Material

Supplementary Figures

## Figures and Tables

**Table 1:** Structural parameters of LPS assuming spherical micelles (radius R_1_) and unilamellar vesicles (radius R_2_) and lamellae thickness.

LPS in D_2_O, pH7	R_1_ (±5)/Å	R_2_ (±10)/Å	Thickness (±5)/Å
LPS, no salt^†^	n/a	1150	40
1 mM NaCl	22	710	45
1 mM NaCl + OligoG	21	709	45
10 mM NaCl	22	710	46
10 mM NaCl + OligoG	22	709	46
100 mM NaCl	23	710	46
100 mM NaCl + OligoG	21	712	46
10 mM NaCl + OligoG, 5 mM Ca^2+†^	n/a	1150	45
10 mM NaCl + OligoG, 10 mM Ca^2+†^	n/a	1150	45
100 mM NaCl + OligoG, 5 mM Ca^2+†^	n/a	1150	43
100 mM NaCl + OligoG, 10 mM Ca^2+†^	n/a	1150	48

The mass fraction and composition of materials have been constrained to physically reasonable values in the fitting routine. The concentrations of LPS and OligoG CF-5/20 were 10 and 20 mg/ml, respectively. ^†^A vesicular structure model, looking at the absolute scattering intensities, was utilised (Bello *et al*. 2014).
