# Supplementary material for: The antimicrobial effects of the alginate oligomer OligoG CF-5/20 are independent of direct bacterial cell membrane disruption
Source: Sci Rep. 2017 Mar 31;7:44731. doi: 10.1038/srep44731 (PMC5374485; doi:10.1038/srep44731)
Supplement: Supplementary Figures [file srep44731-s1.pdf]

The antimicrobial effects of the alginate oligomer OligoG CF-5/20 are independent of direct bacterial cell membrane disruption

Manon F. Pritchard*[1]; Lydia C. Powell[1]; Saira Khan[1]; Peter C. Griffiths[2]; Omar T. Mansour[2]; Ralf Schweins[3]; Konrad Beck[1]; Niklaas J. Buurma[4]; Christopher E. Dempsey[5]; Chris J. Wright[6]; Philip D. Rye[7]; Katja E. Hill[1]; David W. Thomas[†1]; Elaine L. Ferguson[†1]

[1]Advanced Therapies Group, Oral and Biomedical Sciences, School of Dentistry, College of Biomedical and Life Sciences, Cardiff University, Heath Park, Cardiff, UK. [2]Department of Pharmaceutical, Chemical and Environmental Sciences, Faculty of Engineering and Science, University of Greenwich, Medway Campus, Central Avenue, Chatham Maritime, UK. [3]Institut Laue-Langevin, DS/LSS group, 6 rue Jules Horowitz, 38042 Grenoble Cedex 9, France. [4] Physical Organic Chemistry Centre, School of Chemistry, Cardiff University, Cardiff, UK. [5]School of Biochemistry, Biomedical Sciences Building, University Walk, Clifton, BS8 1TD, UK. [6]Centre for NanoHealth, Systems and Process Engineering Centre, College of Engineering, Swansea University, Swansea, UK. [7]AlgiPharma AS, Sandvika, Norway.

*Corresponding author (email: PritchardMF@cardiff.ac.uk). †These authors jointly supervised this work.

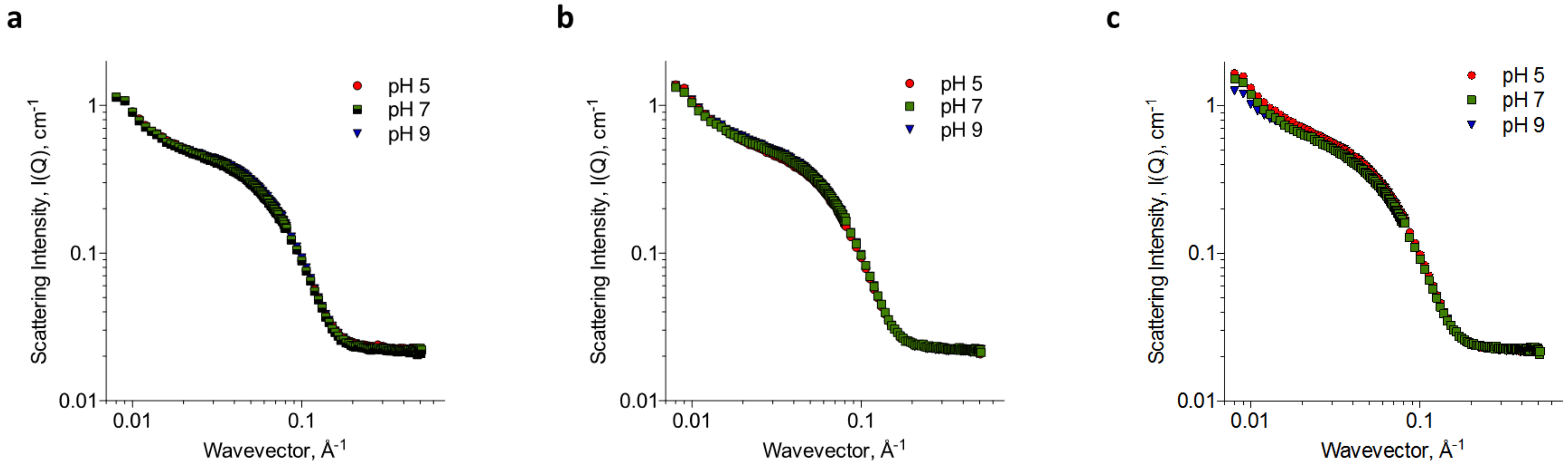

Supplementary Fig. S1. Structural analysis of LPS. Small-angle neutron scattering from LPS (10 mg/ml) in D_2O containing (**a**) 0.001 M NaCl, (**b**) 0.01 M NaCl and (**c**) 0.1 M NaCl at pH 5-9.

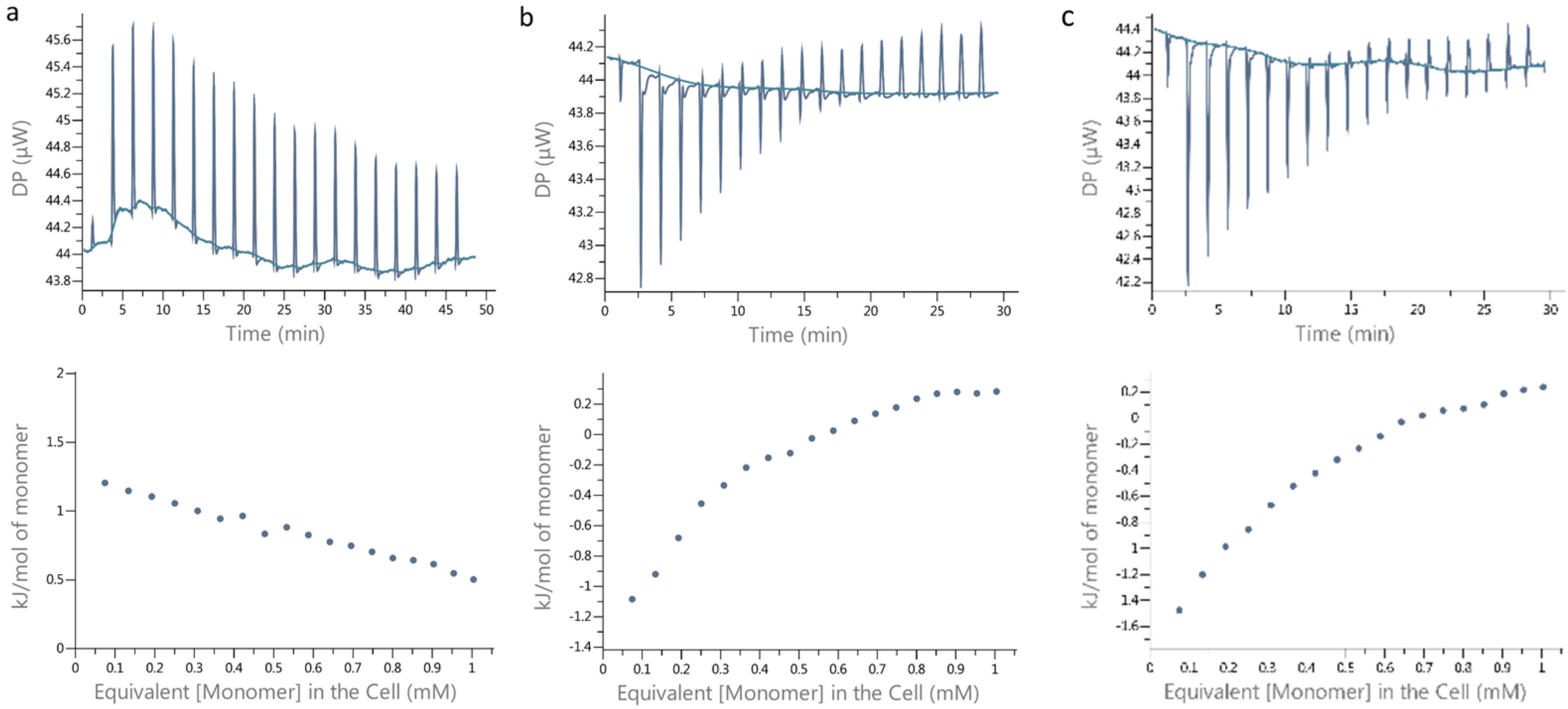

Supplementary Fig. S2. Self-aggregation of OligoG CF-5/20. Enthalpogram for the dilution of 20 mg/ml OligoG CF-5/20 at 37°C in buffer (**a**) 20 mM phosphate pH 7, 100 mM NaCl, 1 mM EDTA, or (**b**) in 20 mM phosphate pH 7, 100 mM NaCl, 1 mM $CaCl_2$ (**c**) or in 20 mM phosphate pH 7, 100 mM NaCl, 1 mM EDTA, 2 mM $CaCl_2$.

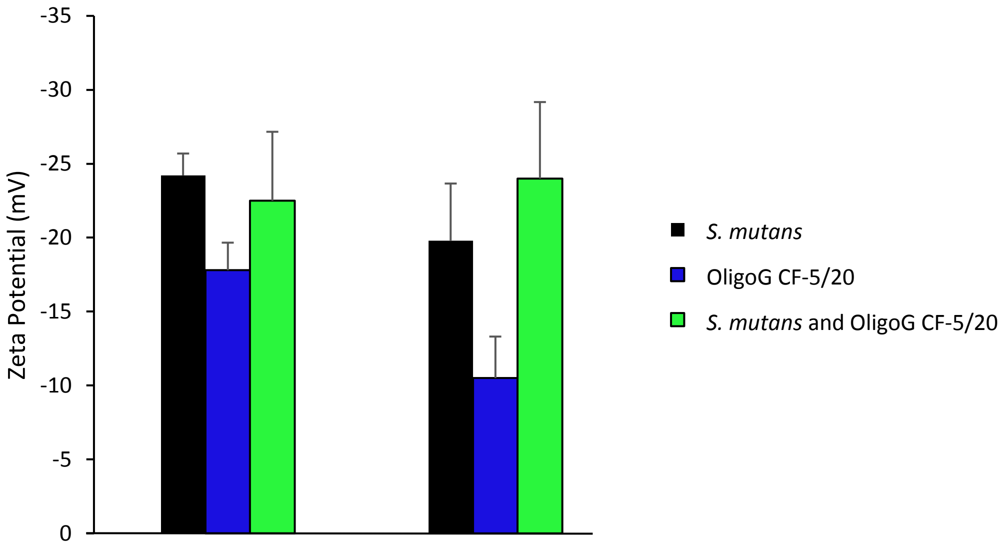

Supplementary Fig. S3. Effect of OligoG CF-5/20 on *S. mutans* cell surface charge at pH 5 and pH 7 (2.6 mM NaCl ± 60 mg/ml OligoG CF-5/20). Mean zeta potential measurements (mV) of *S. mutans*, 60 mg/ml OligoG CF-5/20 and both combined following hydrodynamic shear.
